# Discovery of New Heterocyclic/Benzofuran Hybrids as Potential Anti-Inflammatory Agents: Design, Synthesis, and Evaluation of the Inhibitory Activity of Their Related Inflammatory Factors Based on NF-κB and MAPK Signaling Pathways

**DOI:** 10.3390/ijms24043575

**Published:** 2023-02-10

**Authors:** Yangling Chen, Rui Chen, Renyikun Yuan, Lini Huo, Hongwei Gao, Youqiong Zhuo, Xinxin Chen, Chenwei Zhang, Shilin Yang

**Affiliations:** 1 College of pharmacy, Guangxi University of Chinese Medicine, Nanning 530222, China; 2 Guangxi Key Laboratory of Zhuang and Yao Medicine, Guangxi University of Chinese Medicine, Nanning 530222, China

**Keywords:** benzofuran derivatives, piperazine, anti-inflammatory, NF-κB/MAPK signaling pathway, endotoxemic mice

## Abstract

NF-κB and MAPK are classic inflammation signaling pathways which regulate inflammation signal transmission and induce the expression of many inflammatory factors. Based on the potent anti-inflammatory activity of benzofuran and its derivatives, several new heterocyclic/benzofuran hybrids were first designed and synthesized by molecular hybridization. Their structure was confirmed by ^1^H NMR, ^13^C NMR, HRMS or X-single crystal diffraction. The anti-inflammatory activity of these new compounds was screened by compounds; compound **5d** exhibited an excellent inhibitory effect on the generation of NO (IC_50_ = 52.23 ± 0.97 μM), and low cytotoxicity (IC_50_ > 80 μM) against the RAW-264.7 cell lines. To further elucidate the possible anti-inflammatory mechanisms of compound **5d**, the hallmark protein expressions of the NF-κB and MAPK pathways were studied in LPS-stimulated RAW264.7 cells. The results indicate that compound **5d** not only significantly inhibits the phosphorylation levels of IKKα/IKKβ, IKβα, P65, ERK, JNK and P38 in the classic MAPK/NF-κB signaling pathway in a dose-dependent manner, but also down-regulates the secretion of pro-inflammatory factors such as NO, COX-2, TNF-α and IL-6. Further, the in vivo anti-inflammatory activity of compound **5d** indicated that it could regulate the involvement of neutrophils, leukocytes and lymphocytes in inflammation processes, and reduce the expression of IL-1β, TNF-α and IL-6 in serum and tissues. These results strongly suggest that the piperazine/benzofuran hybrid **5d** has a good potential for developing an anti-inflammatory lead compound, and the anti-inflammatory mechanism might be related to the NF-κB and MAPK signaling pathways.

## 1. Introduction

**Figure 1:**
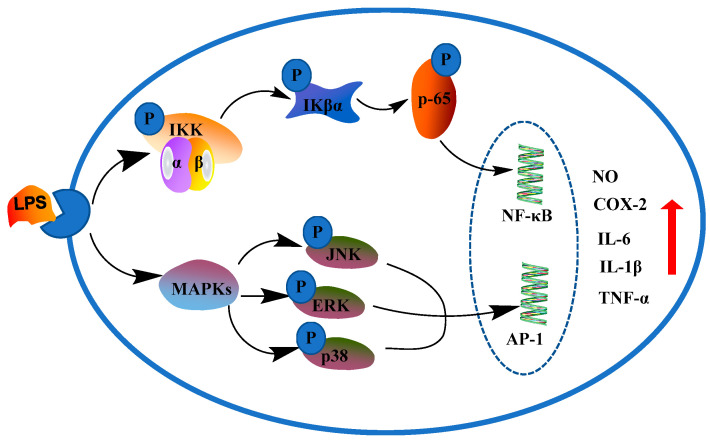
NF-κB and MAPK inflammatory pathways.

Inflammation [1,2] is usually mediated through different mediators resulting in tissue damage and cellular injury. The NF-κB [3,4,5] and MAPK [6,7,8] are classic inflammation signaling pathways which regulate inflammation signal transmission and induce the expression of many inflammatory factors. As shown in Figure 1, some inflammatory mediators such as NO, TNF-α, IL-1β, IL-6 and adhesion molecules are released by activating these two signaling pathways after long-term inflammation [9,10]. Non-steroidal anti-inflammatory drugs (NSAIDs) [11,12] are the most widely used drugs for treating pain and inflammation, but their serious side effects such as bleeding, ulcer, perforation, obstruction, etc., have become obstacles to further applications [13]. Thus, there is a need for identifying and evaluating safer anti-inflammatory drugs.

**Figure 2:**
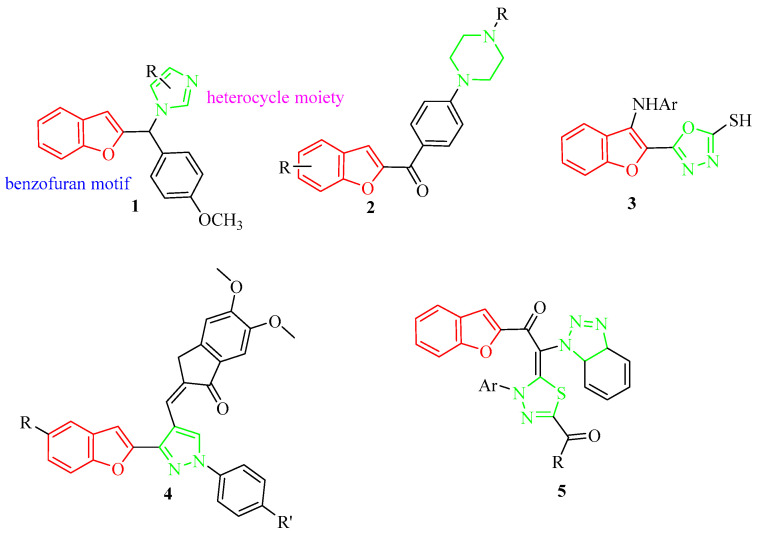
Chemical structures of some benzofuran-heterocycle hybrids.

Benzofuran compounds are a class of compounds that are ubiquitous in nature, which possess a wide range of biological applications, such as antioxidant [14], anti-inflammatory [15], antitumor [16], antibacterial [17], antiviral [18], antituberculosis [19], antidiabetic [20] and antidepression [21,22], etc. Various nitrogen containing heterocyclic fragments such as piperazine, tetrazole and imidazole were reported to exhibit anti-inflammatory properties [23,24,25,26]. Hence, several benzofuran ring systems containing N-heterocyclic moieties displayed excellent anti-inflammatory activities and have attracted more attention (see examples indicated in Figure 2). For example, synthetic 2-benzylbenzofurane-imidazole hybrid (**1**) and 2-(4-imidazoyl benzoyl)benzofuran (**2**) were of interest due to their excellent anti-inflammatory effects [27,28]. Benzofuran derivatives bearing oxadiazole (**3**), pyrazole (**4**) and benzotriazole and 1,3,4-thiadiazole (**5**) nuclei were all found to possess good anti-inflammatory activity [27,28,29,30,31]. 

**Figure 3:**
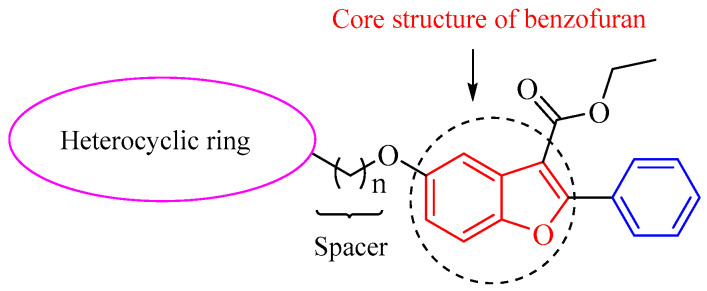
Design strategy for novel hybrid compounds with benzofuran-heterocyclic backbone.

Based on these reports, we have designed and synthesized novel hybrids towards the combination of benzofuran and some nitrogen-containing heterocyclic fragments such as piperazine, tetrazole and imidazole by different chain lengths of the bridge (Figure 3). In the structure, the ester group is retained in the benzofuran skeleton-based prodrugs [32], and different lengths of the bridge (*n* = 3 and *n* = 6) will be chosen for proving whether it can improve the activity of the compouds. In an attempt to find whether the designed compounds are associated with the inflammation signaling pathways of NF-κB and MAPK, their anti-inflammatory activity were screened by LPS-induced RAW264.7 on the generation of NO. Their effect on related inflammatory mediators such as NO, TNF-α, IL-1β, IL-6, etc., were also investigated. In addition, their in vivo anti-inflammatory activities were evaluated by blood routine tests and examination of related inflammatory factors in LPS-reduced endotoxemic mice.

## 2. Results and Discussion

### 2.1. Chemistry

#### Synthetic Route of Benzofuran-Heterocycle Hybrids

**Scheme 1:**
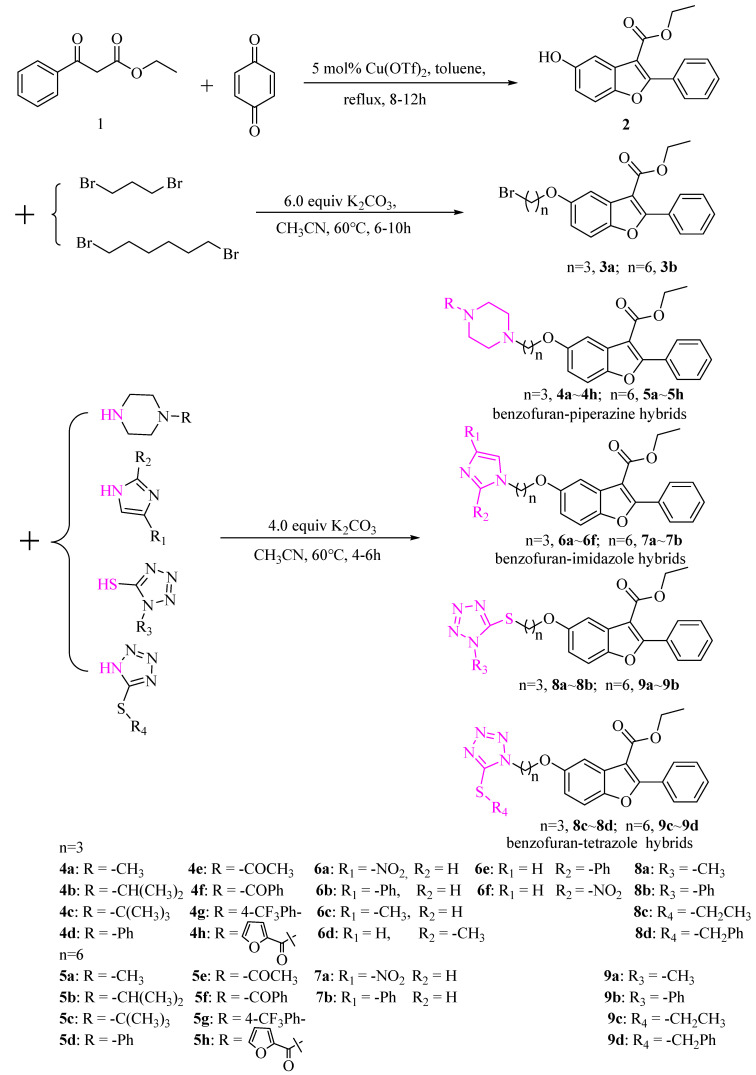
Synthesis of new benzofuran-heterocycle hybrids.

**Figure 4:**
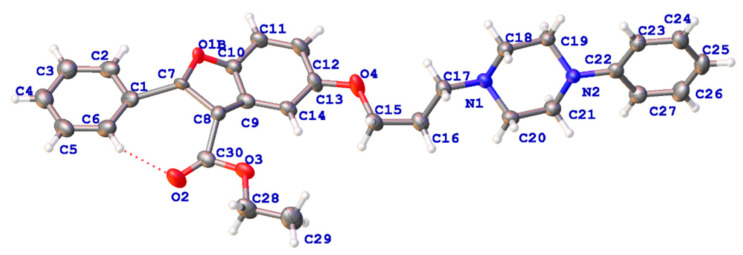
X-ray crystal structure of compound **4d**.

The synthesis of benzofuran-heterocycle hybrids was carried out by the general procedures outlined in Scheme 1. The benzofuran scaffold 2-aryl-5-hydroxy benzofuran ethyl formate (**2**) was constructed according to the Michael addition of ethyl benzoylacetate and p-benzoquinone, followed by cyclization in the reported method [33]. The key intermediate bromides (**3a**, **3b**) were prepared with a 46% or 51% yield of compound **2** with 1,3-dibromopropane or 1,6-dibromohexane. Novel hybrid compounds having a benzofuran–piperazine backbone (**4a–4h**, **5a–5h),** a benzofuran-imidazole backbone (**6a–6f**, **7a–7b**) and a benzofuran–tetrazole backbone (**8a**, **8d**, **9a**, **9d**) were designed and synthesized by the nucleophilic substitution in the presence of potassium carbonate and potassium iodide. The yields of the products were obtained in the ranges 36.8–76%, 36–86% and 82–92.6%, respectively. We found that benzofuran-tetrazole hybrids obtain the highest yield because of the strong nucleophilicity of the sulphur atom in the structure. All compounds were characterized by HRMS, ^1^H NMR and ^13^C NMR. The molecular structure of a representative compound **4d** was confirmed unambiguously by single crystal X-ray diffraction studies (CCDC 2184860). The single crystal diffraction structure of compound **4d** is shown in Figure 4.

### 2.2. In Vitro Studies

#### 2.2.1. Determination of Cytotoxicity and Activity In Vitro

**Figure 5:**
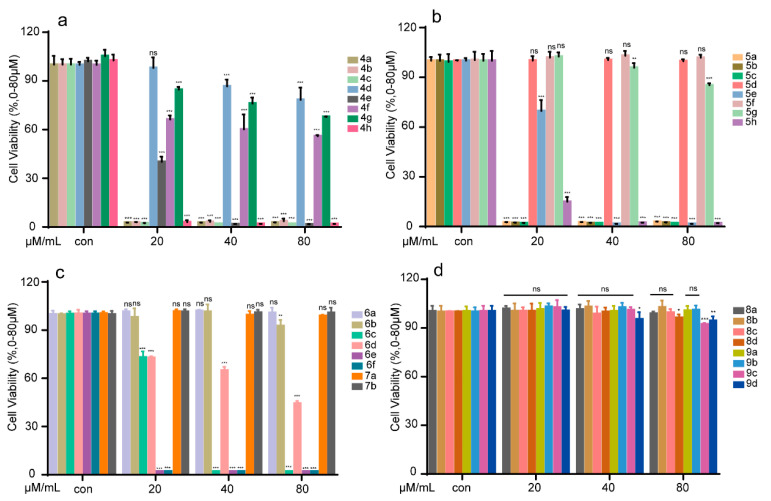
Representation of cell viability assay of benzofuran-heterocycle hybrids **4a**–**4h** (**a**); **5a**–**5h** (**b**); **6a**–**6f** and **7a**–**7b** (**c**); and **8a**–**8d** and **9a**–**9d** (**d**) on RAW264.7. RAW264.7 cells were treated with the compounds (0–80 μM) for 24 h, and the cell viability was detected by MTT assay. All the experiments were independently performed three times. * *p* < 0.05, ** *p* < 0.01, and *** *p* < 0.001 compared to the control group.

RAW 264.7 cells are widely used to establish inflammatory models in vitro. The in vitro anti-inflammatory activities of the synthesized compounds were evaluated using an LPS-stimulated inflammation model, and the results are presented in Figure 5. As expected, most of the benzofuran-heterocycle hybrids (20, 40, 80 μM) displayed no significant toxicity when compared with normal control (con). The benzofuran derivatives containing tetrazole moiety (**8a**, **8d**, **9a–9d**) showed lower cell toxicity at doses of 20–80 μM than other hybrids (Figure 5d), but most of benzofuran derivatives containing piperazine moiety exhibited high toxicity to RAW 264.7 cells except for **4d**, **4g**, **4h**, **5d**, **5f** and **5g** (Figure 5a,b). It is worth mentioning that compound **5d** exhibited no obvious toxic effect on RAW 264.7 cells, and the cell survival rate reached 100%. Compounds **6c, 6e** and **6f** of benzofuran derivatives containing imidazole moiety also showed high cell toxicity (Figure 5c). 

**Figure 6:**
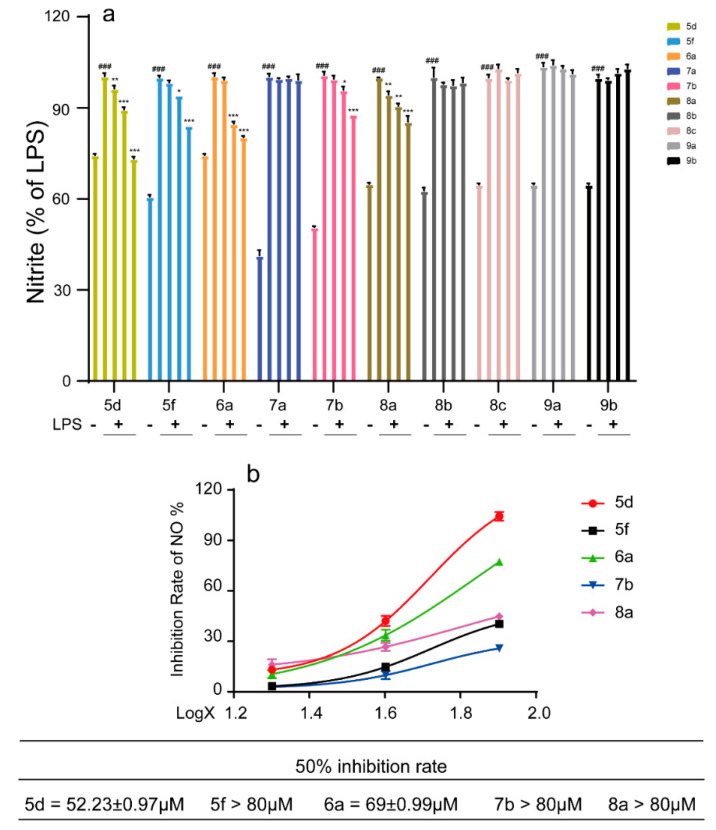
Inhibitory effect of compounds **5d**, **5f**, **6a**, **7a**, **7b**, **8a**–**8c**, **9a**, **9b** (0–80 μM) on over expression of NO in RAW264.7 cells. RAW264.7 cells were treated with the selected compounds for 1 h, and then incubated with LPS for 18 h. The supernatant was collected, and the content of nitrite was detected by Griess assay. All the experiments were independently performed three times. * *p* < 0.05, ** *p* < 0.01, and *** *p* < 0.001 compared with the LPS group; ### *p* < 0.001, compared with control group.

For further investigation of the anti-inflammatory activity of benzofuran-heterocyclic hybrids on generation of NO in LPS-induced RAW 264.7, the low toxicity compounds **5d**, **5f**, **6a**, **7a**, **7b**, **8a–8c**, **9a** and **9b** were then chosen. The results are summarized in Figure 6. Notably, except for the benzofuran-tetrazole hybrids, most of the derivatives had an expected inhibitory effect on the release of NO. Benzofuran–piperazine compounds **5d**, **5f**, **6a**, **7b** and **8a** inhibited the over expression of inflammatory mediator NO in a dose-dependent manner at 20–80 μM (Figure 6b). Especially, the compound **5d** was found to be the most potent anti-inflammatory agent, which could reduce the NO level in LPS-induced RAW 264.7 to normal levels. From the results, most of the tetrazole moiety did not help improve the anti-inflammatory activity of the benzofuran skeleton. Compounds (**8b**, **8c**, **9a** and **9b**) exhibited no obvious inhibitory effect on over expression of NO. 

#### 2.2.2. Effects of Compound **5d** on Pro-Inflammatory Factors in LPS-Induced RAW264.7 Cells

**Figure 7:**
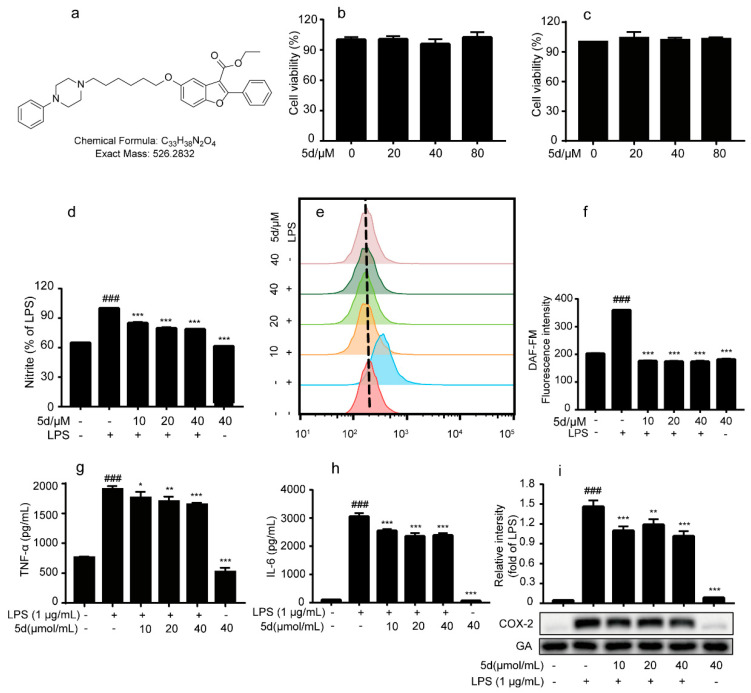
Effects of compound **5d** on pro-inflammatory factors in LPS-induced RAW264.7 cells. (**a**) The chemical structure of **5d**. (**b**,**c**) RAW264.7 cells were treated by **5d** (20, 40 and 80 μM) for 24 h, and the cell viability was determined by MTT assay. (**d**) RAW264.7 cells were pretreated with **5d** (10, 20 and 40 μM) for 1 h and subsequently co-cultured with LPS (1 μg/mL) for an additional 18 h, and the nitrite was detected by Griess assay. (**e**,**f**) RAW264.7 cells were pretreated with **5d** (10, 20 and 40 μM) for 1 h and subsequently cocultured with LPS (1 μg/mL) for an additional 6 h. The medium was collected, and the levels of NO were detected by Flow Cytometry. (**g,h**) RAW264.7 was treated with compound **5d** (10, 20, 40 μmol/mL) for 1 h and then cultured with LPS (1 μg/mL) for 18 h. The supernatant of RAW264.7 cells was collected and the release levels of inflammatory factors TNF-α and IL-6 in the supernatant were detected by ELISA. (**i**) RAW264.7 cells were treated with **5d** for 1 h and co-cultured with LPS (1 μg/mL) for 18 h. Western blotting assay was used to detect the expression of COX-2. All the experiments were independently performed three times. * *p* < 0.05, ** *p* < 0.01, and *** *p* < 0.001 compared with the LPS group; ^###^ *p* < 0.001, compared with control group.

Besides nitric oxide (NO), LPS can upregulate a large number of inflammatory mediators such as cyclooxygenase-2 (COX-2), tumor necrosis factor α (TNF-α), interleukin 6 (IL-6), etc., in RAW264.7 cells during an inflammatory reaction [34,35]. For exploring whether compound **5d** could exhibit an inflammatory effect through the release of pro-inflammatory cytokines, the NO level, the TNF-α and IL-6 production in the supernatants and COX-2 expressions were determined by a flow cytometry assay, enzyme-linked immunosorbent assay (ELISA) and Western blotting assay, respectively. As shown in Figure 7, compound **5d** could suppress NO release in LPS-stimulated RAW264.7 cells in a dose-dependent manner (20–80 µΜ), with a 46.1% inhibition ratio at 40 μM. In addition, the flow cytometry assay indicated that compound **5d** could diminish NO production at 10 μM (Figure 7e,f). Our ELISA assay results indicated that compound **5d** could attenuate TNF-α and IL-6 levels in a dose-dependent manner (Figure 7g,h). As expected the expression of COX-2 in the LPS group was significantly increased, but it was significantly down-regulated after the compound **5d** intervention (Figure 7i). Overall, these findings indicate that compound **5d** diminished inflammatory responses in LPS-stimulated RAW264.7 cells. 

#### 2.2.3. The Effect of **5d** on NF-κB and MAPKs Pathways in RAW264.7 Cells 

NF-κB and MAPK are classic inflammation signaling pathways which regulate inflammation signal transmission and induce the expression of many inflammatory factors. LPS-stimulated RAW264.7 cells can activate ERK, JNK and P38 in MAPK signaling, thereby affecting the activity of a variety of transcription factors, which can further regulate the expression of various cytokines. The NF-κB signaling pathway is involved in the activation of macrophages, and the intervention of this pathway can effectively inhibit the activation of macrophages and inhibit inflammation. To determine whether compound **5d** could regulate the inflammatory processes through the classic MAPK/NF-κB signaling pathway, RAW264.7 cells were treated with LPS or **5d** +LPS, and the levels of phosphorylation of key proteins JNK, P38 and ERK (the MAPK pathway) and IKK, p65 and IκBα (the NF-κB pathway) were all determined by Western blot.

**Figure 8:**
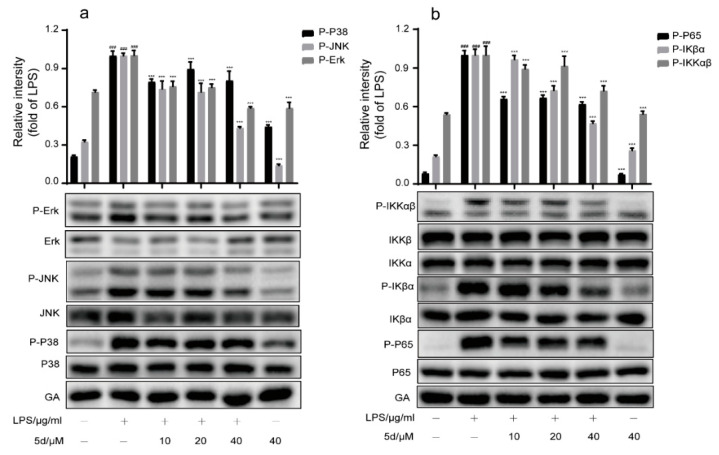
Effect of compound **5d** on MAPK(**a**)/NF-κB(**b**) signaling pathway. (**a,b**) RAW264.7 cells were pretreated with **5d** for 1 h, then co-cultured with LPS (1 μg/mL) for another 6 h. The expression levels of p-ERK, p-P38, p-JNK, p-P65, p-IKβα and p-IKKαβ were determined by Western blotting. All the experiments were independently performed three times. *** *p* < 0.001 compared with the LPS group; ^###^ *p* < 0.001, compared with control group.

**Figure 9:**
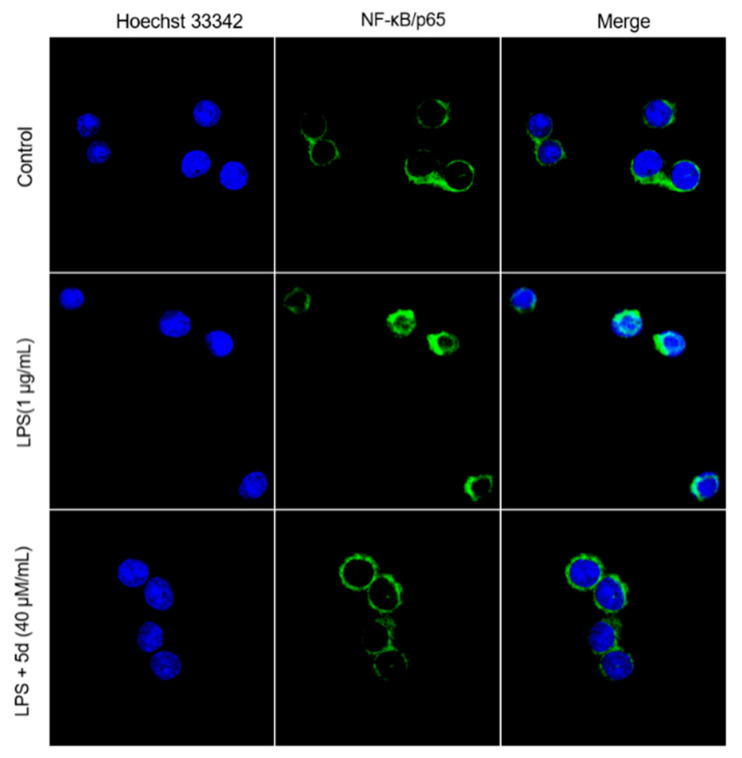
The localization of P65 in the cytoplasm and nucleus of Raw264.7 cells observed by immunofluorescence.

As shown in Figure 8a, the phosphorylation levels of key proteins (JNK, P38 and ERK) were significantly increased in the LPS group when compared to the control group. The phosphorylation levels of JNK, P38 and ERK were inhibited by the **5d** + LPS group. These results suggest that the LPS-stimulated RAW264.7 cells could activate the MAPK pathway, and compound **5d** possessed an excellent anti-inflammatory effect by regulating the expression of P-JNK/P-P38/P-ERK. In addition, the phosphorylation levels of key proteins (p65, IKKα/IKKβ, IKβα) were significantly increased in the LPS group (Figure 8b). As expected, phosphorylation levels of P65, IκBα and IKKα/β were inhibited in a concentration-dependent manner in the **5d** + LPS group (10–40 μM), which were also consistent with the immunofluorescence assay results (Figure 9). These studies demonstrate that, **5d** exhibited anti-infammatory activity through possible NF-κB and MAPKs signaling pathways.

### 2.3. In Vivo Studies

#### 2.3.1. Protective Effect of the Compound **5d** on Endotoxemic Mice 

**Figure 10:**
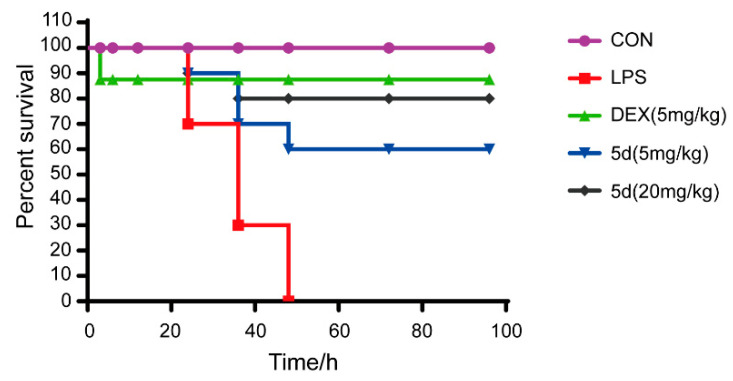
Mouse survival curve of the compound **5d** on endotoxemic mice. Mice in experiment groups were treated with LPS (4 mg/kg, i.p.), and the **5d**-treated groups were injected with **5d** (5, 20 mg/kg, i.p.) at 0, 3, 12, 24, 48 and 72 h after LPS administration. DEX group mice were injected with DEX (5 mg/kg, i.p.) after LPS treatment. The survival rate was recorded for the next 96 h (*n* = 10).

Endotoxemia and related acute lung injury, acute respiratory dysfunction syndrome and multiple organ dysfunction syndrome are the most important reasons for patient infections leading to death. In this study, the endotoxemia mice model was successfully established by intraperitoneal injection of LPS (4 mg/kg) and the therapeutic effects of compound **5d** on endotoxemic mice were investigated. As shown in Figure 10, all endotoxemic mice died within 48 h in the LPS group. Most of the mice injected with two concentrations of **5d** (5 and 20 mg/kg) were alive and in good condition. The mice’s survival rate reached to 90% in the first 24 h and was close to the positive control drug (Dexamethasone, DEX) in the same experimental condition. After 48 h, compound **5d** (5, 20 mg/kg) maintained a high survival rate of 60–80% with a plateau. These results indicate that compound **5d** exhibited a significant protective effect on LPS-induced septic death at doses of 5, 20 mg/kg after 24–96 h.

#### 2.3.2. Effects of **5d** on the Blood Routine and Anti-Inflammatory Cytokines in Serum and Tissue of Endotoxemic Mice

**Figure 11:**
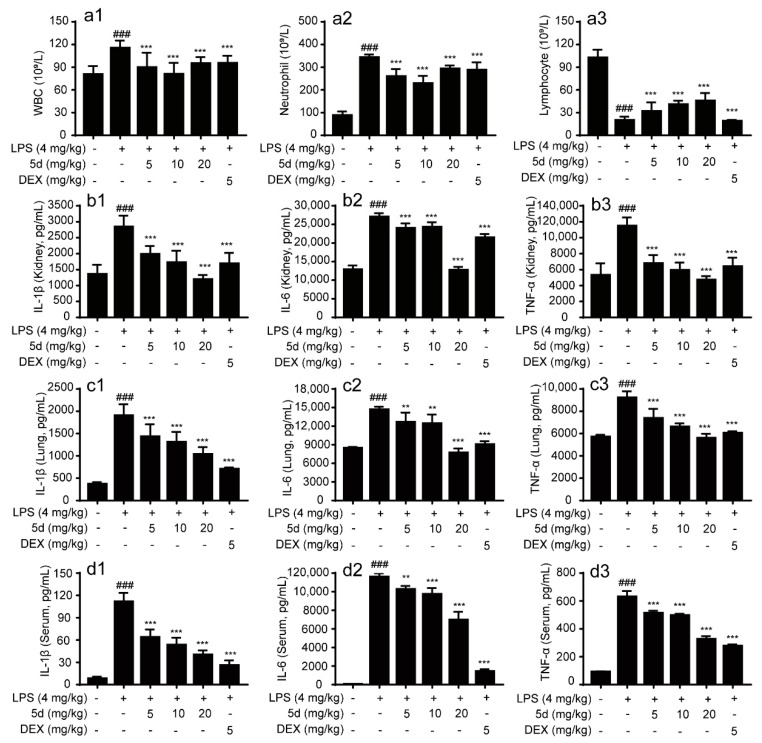
Effects of compound **5d** on serum and different tissues of endotoxemic mice. The mice were intraperitoneally injected with LPS (4 mg/kg) or compound **5d** (5, 10, 20 mg/kg) at the 5th/10th h. The mice were dissected at the 12th h. The changes of WBC (**a1**), NEUT (**a2**), LYM (**a3**), TNF-α, IL-6 and IL-1β in serum (**d1**–**d3**) and tissues (Lung: **c1**–**c3**; Kidney: **b1**–**b3**) were detected. ** *p* < 0.01, and *** *p* < 0.001 compared with the LPS group; ^###^ *p* < 0.001, compared with control group.

Blood routine examination (BRE) is often used for diagnosing related inflammatory disease. During the inflammation, a significant increase in the white blood cells (WBC) and the neutrophils (NEUT) were detected followed by the decrease in lymphocytes (LYM). The blood routine of the endotoxemia mice model proved that an inflammatory response was successfully triggered (Figure 11(a1–a3)). After injection of compound **5d**, the proportion of WBC and NEUT had decreased significantly in the blood routine examination compared with LPS group. It is important to note that the proportion of LYM had gradually increased with the increasing concentration of compound **5d** (5–20 mg/kg). It is thus inferred that compound **5d** participated in the regulation of the inflammation process and affected the blood cell index in endotoxemia mice models.

To further study the effects of **5d** on some inflammatory factors in the serum and tissue of endotoxemic mice, the levels of inflammatory factors such as IL-1β, IL-6 and TNF-α in them were determined by radioimmunoassay. As illustrated in Figure 11b1–d3, the levels of inflammatory cytokines of IL-1β, IL-6 and TNF-α in the model group were significantly higher than those of in the control group (*p* < 0.001). After treatment with compound **5d** (5–20 mg/kg) and DEX (5 mg/kg), all these indexes in the serum, lung and kidney of model rats were remarkably down-regulated in a concentration-dependent manner (*p* < 0.01 or *p* < 0.05). These data show that compound **5d** was more effective in the tissue of the endotoxemic mice, particularly in kidney tissues at high-dose of 20 mg/kg. It is worth mentioning that the levels of inflammatory factors such as IL-6 and TNF-α in lung tissue decreased noticeably after injection of compound **5d**, and even were better than DEX. Based on these results, compound **5d** could alleviate symptoms of inflammation and suppress inflammatory cytokines, especially in lung and kidney tissues of the LPS-induced endotoxemia mice models.

#### 2.3.3. Effects of Compound **5d** on Histopathological Changes of Lung and Kidney Tissues in the Endotoxemia Mice Model

**Figure 12:**
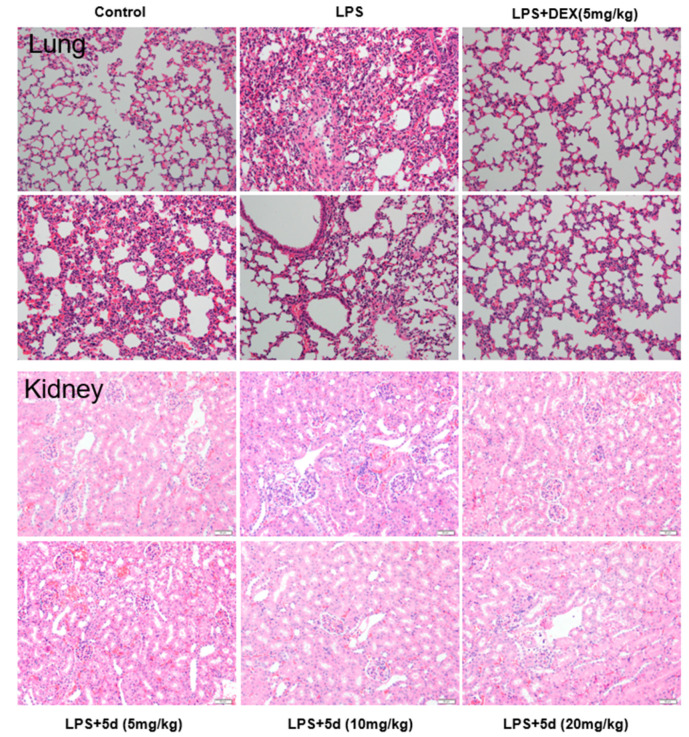
Effect of compound 5d on histopathological changes of lung and kidney tissues in the endotoxemia mice model (*n* = 10). Mice were intraperitoneally injected with LPS (4 mg/kg), then intraperitoneally injected with compound **5d** (5, 10, 20 mg/kg) at the 0th/5th/10th h, dissected at the 12th h. The fixed sections of the tissues and histopathological changes of the tissues were assessed by H and E staining (200×).

To further prove whether compound **5d** could attenuate LPS-induced damage, observational methods and histopathological analysis of the lung and kidney tissues were performed. After 12 h of LPS induction, the lung volume of BALB/c mice in the LPS group had increased. The surface of BALB/c mice indicated a large degree of hyperemia and edema. After administration of compound **5d**, the increase in lung volume of BALB/c mice was not found. The hyperemia and edema observed in the surface was in varying degrees. The size of the kidney showed no obvious changes, and its color was dark red in the LPS or **5d** group. The H and E staining results of lung and kidney tissues were shown in Figure 12. In the blank group, the lung tissue structure of BALB/c mice was normal, no inflammatory cell infiltration was found in the alveoli and the peritracheal space. The bronchiolar walls and the alveoli structure were all intact. After stimulation by LPS, lung tissue structure of BALB/c mice in LPS group was abnormal compared to the blank group. The normal alveolar structure was destroyed, and alveolar interstitial exudation was evident. A large number of infiltrated inflammatory cells were observed in the lung tissue. After intervention by compound **5d** at different doses (5, 10, 20 mg/kg) or with positive control (5 mg/kg), varying extents of improvement in the pathological changes of lung tissues were achieved, including lung interstitial thickening, reduction in pulmonary edema and inflammatory cell infiltration when compared to the LPS group. Compound **5d** could improve the pathological changes of kidney tissues, such as the reduction in infiltration of glomerular inflammatory cells. Low doses of compound **5d** (5 mg/kg) had no obvious improvement in the kidney tissues.

## 3. Materials and Methods 

### 3.1. Chemistry 

All reagents used were commercially obtained. Reactions were monitored by thin-layer chromatography (TLC) on glass plates coated with silica gel using a fluorescent indicator (GF254, Merck, Germany). The NMR spectral data were recorded on Bruker DRX-600 (^1^H: 600 MHz, ^13^C: 151 MHz), respectively. HRMS was performed on an Agilent LC/MSD TOF instrument. All chemical reagents were purchased from Aladdin and Macklin.

#### 3.1.1. Synthesis of Benzofuran-Heterocycle Hybrids

First, the 2-aryl-5-hydroxy benzofuran ethyl formate (**2**) was constructed using ethyl benzoylacetate and p-benzoquinone as starting material according to the reported method [33]. Second, intermediates **3a** and **3b** were prepared by mixing compound **2** (1.0 mmol), 1,3- dibromopropane or 1, 6-dibromohexane (3.0 mmol), anhydrous potassium carbonate (6.0 mmol) in 30 mL acetonitrile and then refluxing for 6–10 h (monitored by TLC). After finishing the reaction, 30 mL of ultrapure water were added, extracted three times by ethyl acetate (30 mL × 3), combined with the organic phase and dried overnight by anhydrous sodium sulfate. After filtration and distilling the solvent, the residue was separated by column chromatography to give pure **3a** and **3b**. Lastly, intermidates **3a** and **3b** (1.0 mmol) with nitrogen-containing heterocyclic compounds (1.2 mmol) such as derivatives of piperazine, mercaptotetrazole, mercaptothiazoline, etc., were added in 20 mL of acetonitrile with anhydrous potassium carbonate (4.0 mmol) and KI (5.0% mmol), and the resulting mixture was stirred continuously for about 4–6 h at 60 °C (monitored by TLC). Then, the mixture was cooled to room temperature and filtered, and the resulting solution was extracted by ethyl acetate (3 × 20 mL) and dried over anhydrous sodium sulfate. After filtration, the solvent was evaporated in vacuum to obtain the crude product and then subjected to column chromatography on silica gel to obtain pure benzofuran-piperazine derivatives (**4a–4h**, **5a–5h**), benzofuran-imidazole derivatives (**6a–6f**, **7a,7b**) and benzofuran-tetrazole derivatives (**8a–8d**, **9a**, **9d**). 

**4a:** Yellow oil; yield 51%; HRMS (*m*/*z*): calculated for C_25_H_30_N_2_O_4_ [M+H]^+^: 423.2283. ^1^H NMR (600 MHz, CDCl_3_) δ: 7.98 (dd, *J* = 6.6, 3.2 Hz, 2H), 7.55 (d, *J* = 7.6 Hz, 1H), 7.51–7.45 (m, 3H), 7.40 (d, *J* = 8.9 Hz, 1H), 6.95 (dd, *J* = 8.9, 2.6 Hz, 1H), 4.40 (q, *J* = 7.1 Hz, 2H), 4.10 (t, *J* = 6.3 Hz, 2H), 2.59–2.50 (m, 10H), 2.30 (s, 3H), 2.05–1.99 (m, 2H), 1.39 (t, *J* = 7.1 Hz, 3H); ^13^C NMR (151 MHz, CDCl_3_) δ: 164.06, 161.33, 156.20, 148.83, 130.16, 129.81, 129.49, 128.01, 127.91, 114.65, 111.61, 108.98, 105.74, 67.09, 60.57, 55.27, 55.16, 53.26, 46.07, 26.91, 14.26.

**4b:** White solid; yield 46%; m.p. 69.5–71.1 °C; HRMS (m/z): calculated for C_27_H_34_N_2_O_4_ [M+H]^+^: 451.2602. ^1^H NMR (600 MHz, CDCl_3_) δ: 7.94 (s, 2H), 7.62–7.47 (m, 5H), 7.03 (d, *J* = 7.1 Hz, 1H), 4.32 (s, 2H), 4.08 (s, 2H), 3.34 (s, 6H), 2.95 (s, 4H), 1.94 (s, 2H), 1.30–1.22 (m, 10H); ^13^C NMR (151 MHz, CDCl_3_) δ: 164.05, 161.31, 156.20, 148.82, 130.15, 129.80, 129.48, 128.00, 127.90, 114.65, 111.59, 108.98, 105.75, 67.14, 60.57, 55.36, 54.46, 53.60, 48.70, 26.91, 18.68, 14.26.

**4c:** White solid; yield 49%; m.p. 80.6–82.6 °C; HRMS (*m*/*z*): calculated for C_28_H_36_N_2_O_4_ [M+H]^+^: 465.2751. ^1^H NMR (600 MHz, CDCl_3_) δ: 7.98 (dd, *J* = 6.0, 2.4 Hz, 2H), 7.55 (d, *J* = 7.9 Hz, 1H), 7.48 (d, *J* = 5.1 Hz, 3H), 7.41 (d, *J* = 8.9 Hz, 1H), 6.95 (dd, *J* = 8.9, 2.1 Hz, 1H), 4.40 (q, *J* = 7.1 Hz, 2H), 4.09 (t, *J* = 6.2 Hz, 2H), 2.64–2.55 (m, 10H), 2.06–2.00 (m, 2H), 1.39 (t, *J* = 7.1 Hz, 3H), 1.09 (s, 9H); ^13^C NMR (151 MHz, CDCl_3_) δ: 164.26, 161.50, 156.36, 148.98, 130.35, 129.96, 129.67, 128.19, 128.06, 114.82, 111.79, 109.13, 105.87, 67.31, 60.76, 55.51, 54.18, 53.91, 45.78, 27.09, 26.04, 14.44.

**4d:** White solid; yield 63%; m.p. 126.5–128.1 °C; HRMS (*m*/*z*): calculated for C_30_H_32_N_2_O_4_ [M+H]^+^: 485.2440. ^1^H NMR (600 MHz, CDCl_3_) δ: 7.94 (s, 2H), 7.57 (dd, *J* = 8.6, 7.8 Hz, 5H), 7.20 (t, *J* = 7.4 Hz, 2H), 7.04 (d, *J* = 8.9 Hz, 1H), 6.93 (d, *J* = 7.8 Hz, 2H), 6.76 (t, *J* = 6.9 Hz, 1H), 4.32 (dd, *J* = 13.6, 6.7 Hz, 2H), 4.10 (s, 2H), 3.34 (s, 4H), 3.13 (s, 4H), 2.54 (s, 2H), 1.97 (s, 2H), 1.30 (t, *J* = 6.8 Hz, 3H); ^13^C NMR (151 MHz, CDCl_3_) δ: 164.06, 161.34, 156.20, 151.35, 148.84, 130.17, 129.81, 129.50, 129.11, 128.01, 127.95, 127.07, 119.67, 116.04, 114.64, 111.63, 108.98, 105.76, 67.03, 60.58, 55.31, 53.34, 49.18, 26.92, 14.27.

**4e:** White solid; yield 59%; m.p. 59.2–61.4 °C; HRMS (*m*/*z*): calculated for C_26_H_30_N_2_O_4_ [M+H]^+^: 451.2263. ^1^H NMR (600 MHz, CDCl_3_) δ: 7.97 (dd, *J* = 6.6, 2.9 Hz, 2H), 7.56 (d, *J* = 7.4 Hz, 1H), 7.49–7.46 (m, 3H), 7.41 (d, *J* = 8.9 Hz, 1H), 6.95 (dd, *J* = 8.9, 2.5 Hz, 1H), 4.39 (q, *J* = 7.1 Hz, 2H), 4.11 (t, *J* = 6.2 Hz, 2H), 3.67–3.61 (m, 2H), 3.54–3.45 (m, 2H), 2.59 (t, *J* = 7.3 Hz, 2H), 2.50–2.47 (m, 4H), 2.10 (s, 3H), 2.06–1.99 (m, 2H), 1.38 (t, *J* = 7.1 Hz, 3H); ^13^C NMR (151 MHz, CDCl_3_) δ: 169.19, 164.26, 161.55, 156.29, 149.01, 130.40, 129.94, 129.69, 128.21, 128.14, 114.77, 111.86, 109.10, 105.79, 66.90, 60.79, 55.26, 53.57, 52.99, 46.46, 41.59, 26.92, 21.55, 14.42.

**4f:** White solid; yield 56%; m.p. 91.5–92.6 °C; HRMS (*m*/*z*): calculated for C_31_H_32_N_2_O_5_ [M+H]^+^: 513.2388. ^1^H NMR (600 MHz, CDCl_3_) δ: 7.97 (dd, *J* = 6.7, 2.9 Hz, 2H), 7.55 (d, *J* = 7.5 Hz, 1H), 7.50–7.46 (m, 3H), 7.42–7.39 (m, 6H), 6.95 (dd, *J* = 8.9, 2.6 Hz, 1H), 4.39 (q, *J* = 7.1 Hz, 2H), 4.11 (t, *J* = 6.3 Hz, 2H), 3.83 (s, 2H), 3.46 (s, 2H), 2.66–2.54 (m, 4H), 2.44 (s, 2H), 2.05–2.00 (m, 2H), 1.38 (t, *J* = 7.1 Hz, 3H); ^13^C NMR (151 MHz, CDCl_3_) δ: 170.57, 164.31, 161.60, 156.36, 149.08, 136.03, 130.46, 130.01, 129.96, 129.76, 128.76, 128.27, 128.21, 127.31, 114.84, 111.92, 109.17, 105.86, 66.94, 60.86, 55.34, 53.87, 53.15, 47.96, 42.36, 26.96, 14.50.

**4g:** White solid; yield 76%; m.p. 154.9–156.7 °C; HRMS (*m*/*z*): calculated for C_31_H_31_F_3_N_2_O_4_ [M+H]^+^: 553.2316. ^1^H NMR (600 MHz, CDCl_3_) δ: 7.97 (dd, *J* = 6.6, 2.9 Hz, 2H), 7.57 (d, *J* = 7.4 Hz, 1H), 7.49 (s, 3H), 7.47 (d, *J* = 6.9 Hz, 2H), 7.42 (d, *J* = 8.9 Hz, 1H), 6.97 (dd, *J* = 8.9, 2.5 Hz, 1H), 6.93 (d, *J* = 8.6 Hz, 2H), 4.39 (q, *J* = 7.1 Hz, 2H), 4.14 (t, *J* = 6.2 Hz, 2H), 3.35–3.28 (m, 4H), 2.68–2.62 (m, 6H), 2.14–2.01 (m, 2H), 1.38 (t, *J* = 7.1 Hz, 3H); ^13^C NMR (151 MHz, CDCl_3_) δ: 164.06, 161.36, 156.19, 153.32, 148.86, 130.19, 129.81, 129.51, 128.02, 127.99, 127.09, 126.40, 126.37, 114.63, 114.46, 111.65, 108.97, 105.73, 66.92, 60.58, 55.21, 53.02, 48.00, 26.89, 14.24.

**4h:** White solid; yield 42%, m.p. 89.9–91.6 °C; HRMS (*m*/*z*): calculated for C_29_H_30_N_2_O_6_ [M+H]^+^: 503.2181. ^1^H NMR (600 MHz, CDCl_3_) δ: 8.08–7.91 (m, 2H), 7.56 (s, 1H), 7.48 (d, *J* = 6.8 Hz, 4H), 7.42 (d, *J* = 8.9 Hz, 1H), 7.00 (d, *J* = 7.0 Hz, 1H), 6.97–6.94 (m, 1H), 6.48 (d, *J* = 1.5 Hz, 1H), 4.40 (q, *J* = 7.1 Hz, 2H), 4.12 (t, *J* = 6.1 Hz, 2H), 3.85 (s, 4H), 2.62 (t, *J* = 7.2 Hz, 2H), 2.56 (s, 4H), 2.05 (q, *J* = 6.5 Hz, 2H), 1.39 (t, *J* = 7.1 Hz, 3H); ^13^C NMR (151 MHz, CDCl_3_) δ: 164.05, 161.35, 159.06, 156.11, 148.84, 147.93, 143.64, 130.18, 129.77, 129.50, 128.00, 127.96, 116.34, 114.59, 111.65, 111.25, 108.93, 105.64, 66.73, 60.58, 55.08, 26.71, 14.23.

**5a:** White solid; yield 56.3%; m.p. 49.8–52.1 °C; HRMS (*m*/*z*): calculated for C_28_H_36_N_2_O_4_ [M+H]^+^: 465.2750. ^1^H NMR (600 MHz, CDCl_3_) δ: 8.00–7.94 (m, 2H), 7.54 (s, 1H), 7.50–7.45 (m, 3H), 7.40 (d, *J* = 8.9 Hz, 1H), 6.95 (dd, *J* = 8.9, 2.2 Hz, 1H), 4.39 (q, *J* = 7.1 Hz, 2H), 4.03 (t, *J* = 6.4 Hz, 2H), 2.46 (s, 6H), 2.38–2.32 (m, 3H), 2.28 (s, 2H), 1.85–1.81 (m, 2H), 1.76 (s, 6H), 1.39 (t, *J* = 7.1 Hz, 5H); ^13^C NMR (151 MHz, CDCl_3_) δ: 163.98, 161.20, 156.14, 148.64, 130.04, 129.66, 129.36, 127.89, 127.75, 114.55, 111.50, 108.82, 105.44, 68.48, 60.46, 58.56, 54.94, 53.08, 45.89, 29.17, 27.30, 26.72, 25.96, 14.12.

**5b:** White solid; yield 48%; m.p. 56.9–57.1 °C; HRMS (*m*/*z*): calculated for C_30_H_40_N_2_O_4_ [M+H]^+^: 493.3064. ^1^H NMR (600 MHz, CDCl_3_) δ: 7.99 (d, *J* = 4.2 Hz, 2H), 7.54 (s, 1H), 7.49 (dd, *J* = 8.4, 6.0 Hz, 3H), 7.41 (d, *J* = 8.9 Hz, 1H), 6.95 (dd, *J* = 8.9, 2.5 Hz, 1H), 4.40 (q, *J* = 7.1 Hz, 2H), 4.04 (t, *J* = 6.5 Hz, 2H), 2.65 (dt, *J* = 13.1, 6.5 Hz, 2H), 2.45–2.27 (m, 6H), 2.13–1.56 (m, 13H), 1.56–1.47 (m, 2H, -CH_2_), 1.39 (t, *J* = 7.1 Hz, 5H); ^13^C NMR (151 MHz, CDCl_3_) δ: 164.08, 161.31, 156.31, 148.79, 130.14, 129.83, 129.49, 128.01, 127.90, 114.70, 111.60, 108.99, 105.65, 68.66, 60.56, 58.76, 54.46, 53.55, 48.65, 29.31, 27.45, 26.86, 26.09, 18.65, 14.25.

**5c:** White solid; yield 36.8%; m.p. 71.3–91.9 °C; HRMS (*m*/*z*): calculated for C_31_H_42_N_2_O_4_ [M+H]^+^: 507.3219. ^1^H NMR (600 MHz, CDCl_3_) δ: 7.98 (d, *J* = 8.7 Hz, 2H), 7.54 (s, 1H), 7.50–7.46 (m, 3H), 7.41 (dd, *J* = 8.9, 2.1 Hz, 1H), 6.95 (d, *J* = 8.9 Hz, 1H), 4.40 (dd, *J* = 7.1, 2.1 Hz, 2H), 4.04 (t, *J* = 5.2 Hz, 2H), 2.58 (d, *J* = 54.5 Hz, 8H), 2.37–2.32 (m, 2H), 1.80 (s, 4H), 1.54 (s, 2H), 1.39 (td, *J* = 7.0, 2.0 Hz, 5H), 1.08 (s, 9H); ^13^C NMR (151 MHz, CDCl_3_) δ: 163.77, 160.98, 155.93, 148.42, 129.82, 129.45, 129.15, 127.67, 127.53, 114.34, 111.28, 108.61, 105.24, 68.28, 60.24, 58.36, 53.57, 53.42, 45.18, 28.96, 27.12, 26.51, 25.75, 25.48, 13.91.

**5d:** White solid; yield 66.8%; m.p. 85.4–86.6 °C; HRMS (*m*/*z*): calculated for C_33_H_38_N_2_O_4_ [M+H]^+^: 527.2913. ^1^H NMR (600 MHz, CDCl_3_) δ: 8.01–7.95 (m, 2H), 7.55 (s, 1H), 7.48 (d, *J* = 5.0 Hz, 3H), 7.41 (d, *J* = 8.9 Hz, 1H), 7.28 (s, 1H), 7.25 (s, 1H), 6.95 (t, *J* = 9.1 Hz, 3H), 6.85 (t, *J* = 7.2 Hz, 1H), 4.40 (q, *J* = 7.1 Hz, 2H), 4.05 (t, *J* = 6.3 Hz, 2H), 3.21 (d, *J* = 4.2 Hz, 4H), 2.62 (s, 4H), 2.45–2.39 (m, 2H), 1.88–1.82 (m, 2H), 1.61–1.58 (m, 2H), 1.57–1.53 (m, 2H), 1.46–1.42 (m, 2H), 1.39 (t, *J* = 7.1 Hz, 3H); ^13^C NMR (151 MHz, CDCl_3_) δ: 164.02, 161.25, 156.21, 151.27, 148.70, 130.10, 129.72, 129.42, 129.03, 127.94, 127.82, 119.58, 115.95, 114.60, 111.56, 108.88, 105.50, 68.53, 60.50, 58.66, 53.26, 49.06, 29.23, 27.34, 26.80, 26.02, 14.18.

**5e:** White solid; yield 66%; m.p. 89.4–90.1 °C; HRMS (*m*/*z*): calculated for C_29_H_36_N_2_O_5_ [M+H]^+^: 493.2706. ^1^H NMR (600 MHz, CDCl_3_) δ: 7.97 (d, *J* = 7.9 Hz, 2H), 7.54 (s, 1H), 7.47 (d, *J* = 4.9 Hz, 3H), 7.41 (d, *J* = 8.9 Hz, 1H), 6.94 (d, *J* = 8.9 Hz, 1H), 4.39 (q, *J* = 7.0 Hz, 2H), 4.04 (t, *J* = 6.2 Hz, 2H), 3.62 (s, 2H), 3.49–3.43 (m, 2H), 2.42 (dd, *J* = 9.0, 5.0 Hz, 4H), 2.39–2.34 (m, 2H), 2.08 (s, 3H), 1.85–1.81 (m, 2H), 1.58–1.49 (m, 6H), 1.38 (t, *J* = 7.1 Hz, 3H); ^13^C NMR (151 MHz, CDCl_3_) δ: 169.12, 164.28, 161.52, 156.44, 148.96, 130.37, 129.97, 129.68, 128.20, 128.10, 114.84, 111.82, 109.12, 105.72, 68.75, 60.76, 58.69, 53.55, 53.00, 46.44, 41.55, 29.48, 27.47, 26.92, 26.24, 21.55, 14.43.

**5f:** Yellow oil; yield 46%; HRMS (*m*/*z*): calculated for C_34_H_38_N_2_O_5_ [M+H]^+^: 555.2856. ^1^H NMR (600 MHz, CDCl_3_) δ: 8.01–7.93 (m, 2H), 7.54 (d, *J* = 7.6 Hz, 1H), 7.49–7.45 (m, 3H), 7.40 (d, *J* = 7.0 Hz, 6H), 6.97–6.91 (m, 1H), 4.42–4.35 (m, 2H), 4.03 (dd, *J* = 8.1, 4.6 Hz, 2H), 3.80 (s, 2H), 3.43 (s, 2H), 2.53 (s, 2H), 2.43–2.33 (m, 4H), 1.88–1.79 (m, 2H), 1.60–1.49 (m, 6H), 1.46–1.34 (m, 5H); ^13^C NMR (151 MHz, CDCl_3_) δ: 170.15, 163.97, 161.20, 156.13, 148.64, 135.66, 130.06, 129.66, 129.56, 129.37, 128.36, 127.89, 127.78, 126.92, 115.23, 114.53, 111.51, 108.81, 105.41, 68.43, 60.46, 58.37, 53.45, 52.74, 47.54, 41.94, 29.59, 29.17, 27.14, 26.54, 25.92, 14.12.

**5g:** White solid; yield 56%; m.p. 104.3–105.0 °C; HRMS (*m*/*z*): calculated for C_34_H_37_F_3_N_2_O_4_ [M+H]^+^: 595.2785. ^1^H NMR (600 MHz, CDCl_3_) δ: 7.98 (dd, *J* = 6.5, 2.8 Hz, 2H), 7.55 (d, *J* = 7.3 Hz, 1H), 7.52–7.44 (m, 5H), 7.41 (d, *J* = 8.9 Hz, 1H), 6.98–6.89 (m, 3H), 4.39 (q, *J* = 7.1 Hz, 2H), 4.05 (t, *J* = 6.4 Hz, 2H), 3.35–3.25 (m, 4H), 2.65–2.56 (m, 4H), 2.46–2.38 (m, 2H), 1.89–1.81 (m, 2H), 1.54 (dd, *J* = 15.9, 8.3 Hz, 4H), 1.44 (dd, *J* = 14.7, 7.6 Hz, 2H), 1.39 (t, *J* = 7.1 Hz, 3H); ^13^C NMR (151 MHz, CDCl_3_) δ: 164.08, 161.33, 156.30, 153.34, 148.81, 130.18, 129.82, 129.50, 128.02, 127.95, 126.38, 126.35, 114.69, 114.43, 111.63, 108.98, 105.64, 68.63, 60.56, 58.63, 53.02, 47.97, 29.31, 27.34, 26.85, 26.07, 14.24.

**5h:** White solid; yield 46%, m.p. 202.8–205.2 °C; HRMS (*m*/*z*): calculated for C_32_H_36_N_2_O_6_ [M+H]^+^: 545.2654. ^1^H NMR (600 MHz, CDCl_3_) δ: 7.98 (d, *J* = 7.8 Hz, 2H), 7.55 (d, *J* = 7.3 Hz, 1H), 7.48 (s, 4H), 7.41 (d, *J* = 8.9 Hz, 1H), 6.99 (d, *J* = 3.2 Hz, 1H), 6.95 (dd, *J* = 8.9, 2.5 Hz, 1H), 6.47 (dd, *J* = 3.2, 1.6 Hz, 1H), 4.39 (q, *J* = 7.1 Hz, 2H), 4.05 (t, *J* = 6.4 Hz, 2H), 3.82 (s, 4H), 2.57–2.47 (m, 4H), 2.43–2.36 (m, 2H), 1.91–1.80 (m, 2H), 1.73 (s, 6H), 1.57–1.53 (m, 2H), 1.39 (t, *J* = 7.1 Hz, 3H); ^13^C NMR (151 MHz, CDCl_3_) δ: 164.09, 161.32, 159.06, 156.28, 148.80, 147.93, 143.64, 130.16, 129.81, 129.49, 128.01, 127.92, 116.31, 114.67, 111.62, 111.25, 108.96, 105.62, 68.61, 60.57, 58.48, 29.30, 27.28, 26.68, 26.05, 14.23.

**6a:** White solid; yield 66%, m.p. 102.8–104.8 °C; HRMS (*m*/*z*): calculated for C_23_H_21_N_3_O_6_ [M+H]^+^: 436.1340. ^1^H NMR (600 MHz, CDCl_3_) δ: 8.51 (s, 1H), 7.94 (dd, *J* = 9.6, 6.3 Hz, 3H), 7.63 (d, *J* = 9.0 Hz, 1H), 7.54 (d, *J* = 6.8 Hz, 3H), 7.42 (d, *J* = 6.3 Hz, 1H), 6.99 (dd, *J* = 8.9, 2.4 Hz, 1H), 4.38–4.27 (m, 4H), 4.04 (t, *J* = 5.8 Hz, 2H), 2.38–2.22 (m, 2H), 1.30 (t, *J* = 7.1 Hz, 3H); ^13^C NMR (151 MHz, CDCl_3_) δ: 163.89, 161.65, 155.23, 149.15, 148.32, 136.22, 130.32, 129.64, 129.55, 128.18, 128.04, 119.20, 114.21, 111.98, 108.91, 105.76, 64.15, 60.66, 45.22, 30.45, 14.24.

**6b:** Yellow oil; yield 57%; HRMS (m/z): calculated for C_29_H_26_N_2_O_4_ [M+H]^+^: 467.1969. ^1^H NMR (600 MHz, CDCl_3_) δ: 7.99 (dd, *J* = 5.8, 2.1 Hz, 2H), 7.75 (d, *J* = 7.8 Hz, 2H), 7.54 (s, 2H), 7.50–7.46 (m, 3H), 7.43 (d, *J* = 8.9 Hz, 1H), 7.36 (t, *J* = 7.5 Hz, 2H), 7.23 (d, *J* = 3.3 Hz, 2H), 6.96 (dd, *J* = 8.8, 1.8 Hz, 1H), 4.37 (q, *J* = 7.1 Hz, 2H), 4.24 (t, *J* = 6.8 Hz, 2H), 4.04 (t, *J* = 5.5 Hz, 2H), 2.36–2.24 (m, 2H), 1.36 (t, *J* = 7.1 Hz, 3H); ^13^C NMR (151 MHz, CDCl_3_) δ: 163.93, 161.53, 155.60, 149.02, 142.36, 137.54, 134.04, 130.24, 129.67, 129.51, 128.58, 128.05, 128.02, 126.77, 124.72, 114.82, 114.34, 111.82, 108.93, 105.88, 64.49, 60.61, 43.80, 30.83, 14.22.

**6c:** Yellow oil; yield 52%; HRMS (*m*/*z*): calculated for C_24_H_24_N_2_O_4_ [M+H]^+^: 405.1815. ^1^H NMR (600 MHz, CDCl_3_) δ: 7.98 (dd, *J* = 6.0, 2.4 Hz, 2H), 7.53 (s, 1H), 7.48 (dd, *J* = 5.6, 3.9 Hz, 3H), 7.43 (d, *J* = 9.0 Hz, 1H), 6.95 (dt, *J* = 5.2, 2.4 Hz, 1H), 6.66 (s, 1H), 4.39 (q, *J* = 7.1 Hz, 2H), 4.19–4.08 (m, 2H), 4.00 (t, *J* = 5.6 Hz, 2H), 2.30–2.18 (m, 5H), 1.38 (t, *J* = 7.1 Hz, 3H); ^13^C NMR (151 MHz, CDCl_3_) δ: 163.95, 161.51, 155.65, 149.00, 138.42, 136.86, 136.37, 130.24, 129.51, 128.02, 115.47, 114.35, 111.79, 108.93, 105.79, 64.55, 60.61, 43.49, 41.12, 30.84, 14.24, 13.62.

**6d:** Yellow powder; yield 80%, m.p. 129.4–130.7 °C; HRMS (*m*/*z*): calculated for C_23_H_21_N_3_O_6_ [M+H]^+^: 436.1496. ^1^H NMR (600 MHz, CDCl_3_) δ: 7.99–7.96 (m, 2H), 7.52 (d, *J* = 7.6 Hz, 1H), 7.48 (dd, *J* = 6.5, 2.7 Hz, 3H), 7.43 (d, *J* = 8.9 Hz, 1H), 7.13 (s, 1H), 7.10 (d, *J* = 0.6 Hz, 1H), 6.93 (dd, *J* = 8.9, 2.6 Hz, 1H), 4.71 (t, *J* = 6.7 Hz, 2H), 4.39 (q, *J* = 7.1 Hz, 2H), 4.04 (t, *J* = 5.5 Hz, 2H), 2.42–2.36 (m, 2H), 1.38 (t, *J* = 7.1 Hz, 3H); ^13^C NMR (151 MHz, CDCl_3_) δ: 163.91, 161.61, 155.36, 149.07, 130.30, 129.65, 129.51, 128.45, 128.11, 128.04, 126.58, 114.25, 111.91, 108.94, 105.63, 64.29, 60.66, 47.31, 29.85, 14.27.

**6e:** White solid; yield 61%, m.p. 91.3–93.5 °C; HRMS (*m*/*z*): calculated for C_29_H_26_N_2_O_4_ [M+H]^+^: 467.1970. ^1^H NMR (600 MHz, CDCl_3_) δ: 8.00–7.96 (m, 2H), 7.60 (d, *J* = 7.6 Hz, 2H), 7.48 (dd, *J* = 6.4, 5.0 Hz, 4H), 7.45–7.38 (m, 4H), 7.16 (s, 1H), 7.07 (s, 1H), 6.84 (dd, *J* = 8.8, 1.6 Hz, 1H), 4.39 (q, *J* = 7.1 Hz, 2H), 4.33 (t, *J* = 7.0 Hz, 2H), 3.98 (t, *J* = 5.5 Hz, 2H), 2.28–2.21 (m, 2H), 1.37 (t, *J* = 7.1 Hz, 3H); ^13^C NMR (151 MHz, CDCl_3_) δ: 163.96, 161.48, 155.48, 148.96, 147.84, 130.25, 129.70, 129.51, 128.87, 128.60, 128.03, 120.70, 114.31, 111.74, 108.92, 105.52, 64.42, 60.61, 43.58, 30.75, 14.26.

**6f:** Yellow oil; yield 36%; HRMS (*m*/*z*): calculated for C_24_H_24_N_2_O_4_ [M+H]^+^: 405.1813. ^1^H NMR (600 MHz, CDCl_3_) δ: 7.98 (dd, *J* = 6.1, 2.4 Hz, 2H), 7.53 (d, *J* = 2.4 Hz, 1H), 7.50–7.47 (m, 3H), 7.44 (d, *J* = 8.9 Hz, 1H), 6.95 (dd, *J* = 10.3, 3.6 Hz, 2H), 6.85 (s, 1H), 4.39 (q, *J* = 7.1 Hz, 2H), 4.14 (t, *J* = 6.7 Hz, 2H), 4.00 (t, *J* = 5.5 Hz, 2H), 2.40 (s, 3H), 2.23 (dt, *J* = 12.2, 6.3 Hz, 2H), 1.38 (t, *J* = 7.1 Hz, 3H); ^13^C NMR (126 MHz, CDCl_3_) δ: 164.29, 161.89, 155.85, 149.33, 144.98, 130.60, 130.00, 129.85, 128.37, 127.28, 119.52, 114.62, 112.18, 109.25, 105.98, 77.61, 77.36, 77.11, 64.51, 60.97, 42.83, 30.68, 14.58, 13.08.

**7a:** Yellow powder; yield 78.3%, m.p. 98.7–100.3 °C; HRMS (*m*/*z*): calculated for C_26_H_27_N_3_O_6_ [M+H]^+^: 478.1977. ^1^H NMR (600 MHz, CDCl_3_) δ: 7.97 (dd, *J* = 6.3, 2.8 Hz, 2H), 7.77 (s, 1H), 7.55 (d, *J* = 7.4 Hz, 1H), 7.50–7.46 (m, 3H), 7.45–7.40 (m, 2H), 6.94 (dd, *J* = 8.9, 2.5 Hz, 1H), 4.39 (q, *J* = 7.1 Hz, 2H), 4.04 (dd, *J* = 9.9, 4.1 Hz, 4H), 1.88-1.81 (m, 4H), 1.60–1.54 (m, 2H), 1.44 (dt, *J* = 15.3, 7.8 Hz, 2H), 1.38 (t, *J* = 7.1 Hz, 3H); ^13^C NMR (151MHz, CDCl_3_) δ: 164.15, 161.46, 156.16, 148.90, 135.94, 130.29, 129.83, 129.59, 128.10, 128.06, 119.07, 114.66, 111.79, 108.97, 105.55, 68.24, 60.67, 48.44, 30.76, 29.11, 26.27, 25.71, 14.30.

**7b:** White solid; yield 45.2%, m.p. 52.8–55.2 °C; HRMS (*m*/*z*): calculated for C_32_H_32_N_2_O_4_ [M+H]^+^: 509.2437. ^1^H NMR (600 MHz, CDCl_3_) δ: 8.00–7.95 (m, 2H), 7.78 (d, *J* = 7.8 Hz, 2H), 7.62 (s, 1H), 7.54 (s, 1H), 7.48 (d, *J* = 5.0 Hz, 3H), 7.42–7.35 (m, 3H), 7.25–7.20 (m, 2H), 6.94 (dd, *J* = 8.8, 1.3 Hz, 1H), 4.39 (q, *J* = 7.0 Hz, 2H), 4.02 (dt, *J* = 14.1, 6.6 Hz, 4H), 1.91–1.87 (m, 2H), 1.85–1.82 (m, 2H), 1.57 (dt, *J* = 15.3, 7.8 Hz, 2H), 1.44 (dt, *J* = 15.0, 7.7 Hz, 2H), 1.38 (t, *J* = 7.1 Hz, 3H); ^13^C NMR (151 MHz, CDCl_3_) δ: 164.07, 161.35, 156.16, 148.81, 137.01, 130.18, 129.50, 128.66, 128.01, 126.99, 124.80, 114.62, 111.67, 108.94, 105.55, 68.32, 60.57, 47.43, 30.95, 29.12, 26.34, 25.71, 14.23.

**8a:** White solid; yield 86%, m.p. 124.4–124.7 °C; HRMS (*m*/*z*): calculated for C_22_H_22_N_4_O_4_S [M+H]^+^: 439.1437. ^1^H NMR (600 MHz, CDCl_3_) δ: 7.98 (dd, *J* = 6.5, 3.2 Hz, 2H), 7.56 (d, *J* = 7.6 Hz, 1H), 7.50–7.46 (m, 3H), 7.42 (d, *J* = 8.9 Hz, 1H), 6.96 (dd, *J* = 8.9, 2.6 Hz, 1H), 4.40 (q, *J* = 7.1 Hz, 2H), 4.19 (t, *J* = 5.7 Hz, 2H), 3.93 (s, 3H), 3.60 (t, *J* = 7.0 Hz, 2H), 2.41–2.35 (m, 2H), 1.39 (t, *J* = 7.1 Hz, 3H); ^13^C NMR (151 MHz, CDCl_3_) δ: 163.99, 161.47, 155.80, 154.15, 148.96, 130.23, 129.72, 129.50, 128.03, 127.99, 114.53, 111.76, 108.97, 105.72, 66.30, 60.63, 33.38, 30.11, 29.06, 14.29.

**8b:** White solid; yield 76%, m.p. 112.6–114.5 °C; HRMS (*m*/*z*): calculated for C_27_H_24_N_4_O_4_S [M+H]^+^: 501.1595. ^1^H NMR (600 MHz, CDCl_3_) δ: 8.00–7.97 (m, 2H), 7.57 (dd, *J* = 6.7, 3.4 Hz, 6H), 7.49–7.46 (m, 3H), 7.41 (d, *J* = 8.9 Hz, 1H), 6.94 (dd, *J* = 8.9, 2.6 Hz, 1H), 4.39 (q, *J* = 7.1 Hz, 2H), 4.19 (t, *J* = 5.8 Hz, 2H), 3.64 (t, *J* = 7.0 Hz, 2H), 2.44–2.38 (m, 2H), 1.38 (t, *J* = 7.1 Hz, 3H); ^13^C NMR (151 MHz, CDCl_3_) δ: 163.99, 161.45, 155.80, 154.18, 148.95, 133.67, 130.22, 130.15, 129.82, 129.73, 129.50, 128.03, 127.96, 123.83, 114.55, 111.74, 108.97, 105.72, 66.36, 60.62, 30.14, 28.93, 14.28.

**8c:** Yellow oil; yield 87%; HRMS (*m*/*z*): calculated for C_23_H_24_N_4_O_4_S [M+H]^+^: 453.1599. ^1^H NMR (600 MHz, CDCl_3_) δ: 8.01–7.97 (m, 2H), 7.53 (s, 1H), 7.48 (d, *J* = 7.5 Hz, 3H), 7.42 (d, *J* = 8.9 Hz, 1H), 6.94 (d, *J* = 8.9 Hz, 1H), 4.85 (t, *J* = 6.9 Hz, 2H), 4.40 (q, *J* = 7.1 Hz, 2H), 4.12 (t, *J* = 5.6 Hz, 2H), 3.20 (q, *J* = 7.3 Hz, 2H), 2.57–2.50 (m, 2H), 1.40 (dt, *J* = 14.5, 7.2 Hz, 6H); ^13^C NMR (151 MHz, CDCl_3_) δ: 164.28, 163.98, 161.51, 155.62, 149.04, 130.25, 129.71, 129.52, 128.04, 127.97, 114.56, 111.78, 108.97, 105.85, 64.89, 60.64, 50.43, 29.18, 26.48, 14.96, 14.28.

**8d:** Yellow oil; yield 88.6%; HRMS (*m*/*z*): calculated for C_28_H_26_N_4_O_4_S [M+H]^+^: 515.1754. ^1^H NMR (600 MHz, CDCl_3_) δ: 7.97 (dd, *J* = 5.9, 2.1 Hz, 2H), 7.52 (s, 1H), 7.47 (d, *J* = 5.0 Hz, 3H), 7.41 (d, *J* = 8.9 Hz, 1H), 7.37 (d, *J* = 7.6 Hz, 2H), 7.27 (t, *J* = 7.5 Hz, 2H), 7.23 (t, *J* = 7.3 Hz, 1H), 6.92 (dd, *J* = 8.8, 1.8 Hz, 1H), 4.83 (t, *J* = 6.8 Hz, 2H), 4.41–4.36 (m, 4H, –SCH_2_), 4.07 (t, *J* = 5.6 Hz, 2H), 2.53–2.47 (m, 2H), 1.38 (t, *J* = 7.1 Hz, 3H); ^13^C NMR (151 MHz, CDCl_3_) δ: 163.95, 163.74, 161.48, 155.58, 149.01, 136.51, 130.23, 129.68, 129.50, 129.02, 128.57, 128.01, 127.95, 127.62, 114.52, 111.76, 108.94, 105.84, 64.81, 60.62, 50.41, 36.47, 29.11, 14.25.

**9a:** White solid; yield 92.6%, m.p. 89.6–90.5 °C; HRMS (*m*/*z*): calculated for C_25_H_28_N_4_O_4_S [M+H]^+^: 481.1908. ^1^H NMR (600 MHz, CDCl_3_) δ: 8.02–7.95 (m, 2H), 7.54 (d, *J* = 7.1 Hz, 1H), 7.51–7.45 (m, 3H), 7.41 (d, *J* = 8.9 Hz, 1H), 6.95 (dd, *J* = 8.8, 2.2 Hz, 1H), 4.40 (q, *J* = 7.1 Hz, 2H), 4.04 (t, *J* = 6.3 Hz, 2H), 3.91 (s, 3H), 3.38 (t, *J* = 7.3 Hz, 2H), 1.91–1.81 (m, 4H), 1.57 (d, *J* = 3.4 Hz, 4H), 1.39 (t, *J* = 7.1 Hz, 3H); ^13^C NMR (126 MHz, CDCl_3_) δ: 164.13, 161.38, 156.26, 154.48, 148.84, 130.24, 129.83, 129.55, 128.07, 127.96, 114.68, 111.71, 109.00, 105.60, 68.45, 60.65, 33.39, 33.25, 29.32, 29.22, 28.40, 25.67, 14.32.

**9b:** White solid; yield 76%, m.p. 92.4–93.6 °C; HRMS (*m*/*z*): calculated for C_30_H_30_N_4_O_4_S [M+H]^+^: 543.2068. ^1^H NMR (600 MHz, CDCl_3_) δ: 7.98 (d, *J* = 7.7 Hz, 2H), 7.58 (t, *J* = 6.4 Hz, 4H), 7.54 (d, *J* = 7.1 Hz, 2H), 7.48 (d, *J* = 4.8 Hz, 3H), 7.41 (d, *J* = 8.9 Hz, 1H), 6.94 (d, *J* = 8.8 Hz, 1H), 4.39 (q, *J* = 7.1 Hz, 2H), 4.04 (t, *J* = 6.2 Hz, 2H), 3.43 (t, *J* = 7.2 Hz, 2H), 1.94–1.79 (m, 4H), 1.56 (s, 4H), 1.39 (t, *J* = 7.1 Hz, 3H); ^13^C NMR (126 MHz, CDCl_3_) δ: 163.99, 161.24, 156.12, 154.37, 148.69, 133.63, 130.09, 130.01, 129.71, 129.41, 127.93, 127.81, 123.76, 114.55, 111.56, 108.87, 105.46, 68.32, 60.50, 33.15, 29.08, 28.97, 28.33, 25.55, 14.18.

**9c:** White solid; yield 92%, m.p. 68.2–70.1 °C; HRMS (*m*/*z*): calculated for C_26_H_30_N_4_O_4_S [M+H]^+^: 495.2065. ^1^H NMR (600 MHz, CDCl_3_) δ: 8.00–7.96 (m, 2H), 7.54 (d, *J* = 7.4 Hz, 1H), 7.50–7.46 (m, 3H), 7.41 (d, *J* = 8.9 Hz, 1H), 6.94 (dd, *J* = 8.9, 2.4 Hz, 1H), 4.59 (t, *J* = 7.1 Hz, 2H), 4.40 (q, *J* = 7.1 Hz, 2H), 4.03 (t, *J* = 6.3 Hz, 2H), 3.21 (q, *J* = 7.4 Hz, 2H), 2.09–2.03 (m, 2H), 1.86–1.80 (m, 2H), 1.60–1.54 (m, 4H), 1.43–1.37 (m, 6H); ^13^C NMR (151 MHz, CDCl_3_) δ: 164.06, 164.00, 161.34, 156.17, 148.80, 130.16, 129.78, 129.49, 128.00, 127.90, 114.63, 111.65, 108.96, 105.58, 68.33, 60.57, 53.23, 29.18, 29.08, 26.50, 26.15, 25.53, 14.95, 14.25.

**9d:** White solid; yield 87%, m.p. 64.8–65.6 °C; HRMS (*m*/*z*): calculated for C_31_H_32_N_4_O_4_S [M+H]^+^: 557.2225. ^1^H NMR (600 MHz, CDCl_3_) δ: 8.00–7.96 (m, 2H), 7.54 (s, 1H), 7.48 (d, *J* = 5.1 Hz, 3H), 7.40 (dd, *J* = 13.0, 8.3 Hz, 3H), 7.30 (t, *J* = 7.5 Hz, 2H), 7.25–7.21 (m, 1H), 6.94 (dd, *J* = 8.8, 1.7 Hz, 1H), 4.58 (t, *J* = 7.0 Hz, 2H), 4.40 (dd, *J* = 14.0, 6.9 Hz, 4H), 4.03 (t, *J* = 6.3 Hz, 2H), 2.07–2.00 (m, 2H), 1.85–1.79 (m, 2H), 1.57 (s, 4H), 1.39 (t, *J* = 7.2 Hz, 3H); ^13^C NMR (151 MHz, CDCl_3_) δ: 164.06, 163.49, 161.35, 156.18, 148.81, 136.58, 130.17, 129.49, 129.03, 128.57, 128.01, 127.62, 114.62, 111.66, 108.96, 105.57, 68.33, 60.57, 53.28, 36.53, 29.14, 29.07, 26.11, 25.53, 14.25.

#### 3.1.2. Preparation of Single Crystal Compound **4d** and Its X-ray Single Crystal 

A single crystal suitable for an X-ray diffraction study was cultivated from chloroform and methyl alcohol by a slow evaporation method at room temperature. The colorless block crystal of **4d** had approximate dimensions of 0.12 × 0.11 × 0.09 mm^3^. All measurements were performed with Mo Kα radiation (λ = 0.7103 Å) on a SuperNova, Dual, Cu at zero, AtlasS2 diffractometer and the crystal was kept at 150.00(10) K during data collection. A total of 8435 reflections were collected in the range 4.432° ≤ 2θ ≤ 49.998° (−11 ≤ h ≤ 10, −14 ≤ k ≤ 12, −14 ≤ l ≤ 14) by using the w scan mode; 4450 were independent with Rint = 0.0398, of which 2403 were observed with I > 2σ(I) and used in structure determination and refinements. The structure was derived by direct methods with SHELXS and refined by SHELXL. All non-hydrogen atoms were refined with anisotropic thermal parameters. The hydrogen atoms were placed in the calculated positions. The final full-matrix least-squares refinement gave R = 0.0552, wR = 0.1162, S = 1.036, (Δρ)max = 0.22 and (Δρ)min = −0.20 e/Å3.

### 3.2. Biological Evaluation

#### 3.2.1. Cell Culture

RAW264.7 cells were purchased from the Cell Bank of the Chinese Academy of Sciences (Shanghai, China) and cultured in high-glucose Dulbecco’s modified Eagle’s medium (DMEM; Gaithersburg, MD, USA) containing 10% of fetal bovine serum (FBS; Sigma, St. Louis, MO, USA) and 1% of antibiotic solution. The cells were grown in an incubator with 5% of CO_2_ at 37 °C. All cells used were in the logarithmic growth phase.

#### 3.2.2. MTT Assay

The cell viability was detected by MTT assay. RAW264.7 cells (3.0 × 10^4^ cells/well) were seeded in 96-well culture plates for 12 h. They were then treated with various diluted concentrations of compounds for 24 h, and the control cells were treated with culture medium alone. Then 10 μL of MTT solution (5 mg/mL) was added to each well and cultured for 4 h. Next, the supernatant was discarded and DMSO was added to each well (100 μL/well). The solutions were mixed thoroughly to dissolve MTT-formazan crystals and the absorbance at 570 nm was measured by iMark Microplate Absorbance Reader (BioTek, Winkowski, VT, USA). All the experiments were repeated at least three times.

#### 3.2.3. Griess Assay

The NO content of the cell supernatant was determined by the Griess assay. RAW264.7 cells were inoculated in 12-well plates at a density of 15 × 10^4^ per well and cultured overnight. First, cells were pretreated with different concentrations of drugs for 1 h, and then co-incubated with 1 μg/mL LPS for 18 h. The medium was collected to determine the nitrite level using the Griess assay. Griess reagent (50 µL) was added into the supernatant (50 µL) and incubated at 37 °C for 30 min in the dark. Absorbance was measured at 540 nm using an iMark Microplate Absorbance Reader (BioTek, Winkowski, VT, USA). The concentration of NO was calculated by using 0–100 µM sodium nitrite standards. All the experiments were repeated at least three times.

#### 3.2.4. Flow Cytometry Analysis

RAW264.7 cells were seeded in a 24-well plate at a density of 15 × 10^4^ cells per well. The cells were cultured in DMEM with 10% of FBS and incubated at 37 °C in a 5% CO_2_ atmosphere overnight. Next, cells were pretreated with **5d** (0, 10, 20 and 40 µM) for 1 h, and then co-incubated with 1 μg/mL LPS for 8 h. The supernatant was discarded, and the NO probe (DAF-FM, 1 µM/mL) was added and incubated for 1 h. After removing the medium, the cells were washed with PBS buffer (1 mL) twice and resuspended in PBS. The suspension was filtered with a 200-mesh filter and tested by flow cytometry (Becton-Dickinson, Franklin Lakes, NJ, USA).

#### 3.2.5. Fluorescence Imaging in RAW264.7 Cells by Confocal Microscopy

RAW264.7 cells (6 × 10^5^ cells/dish) were seeded in Confocal dishes (control) and cultured in DMEM with 10% FBS and incubated at 37 °C in a 5% CO_2_ atmosphere overnight. The cells were pre-treated with **5d** (0 or 40 µM) for 1 h, then co-incubated with 1 μg/mL LPS for 2 h. Next, the supernatant was discarded and washed with PBS buffer (1 mL) three times. About 200 μL of 4% paraformaldehyde (PFA) fix solution was added to fix the cells for 30 min at room temperature. Then 200 μL PBS buffer containing 0.10–0.20% Triton X-100 was added to punch for 15 min. They were then treated with 200 μL of PBS buffer containing 5% BSA for 30 min to block non-specific binding of antibodies. The NF-κB p65 antibody was diluted with 5% BSA in PBS and incubated overnight at 4 °C. Finally, cells were incubated with Alexa Fluor 594 secondary antibody for 1 h at room temperature. Nuclei were revealed by Hoechst 33,342 staining. Fluorescence images were collected under a confocal microscope system (Leica, Wetzlar, Germany).

#### 3.2.6. ELISA Assay

RAW264.7 cells (6 × 10^5^ cells/well) were seeded in a 6-well culture plate and cultured overnight. The cells were pre-treated with **5d** (0, 10, 20, and 40 µM) for 1 h, and then co-incubated with 1 μg/mL LPS for 18 h. The supernatant was collected and analyzed by mouse IL-1β/IL-6/TNF-α ELISA Assay Kit.

#### 3.2.7. Western Blotting Analysis

RAW264.7 cells were seeded in a dish or 6-well (6 × 10^5^ cells/well) plate and cultured overnight. The cells were pre-treated with **5d** (0, 10, 20, 40 µM) for 1 h, then LPS (0 or 1 µg/mL) was added to incubate for 6 h. Total cell proteins were harvested using RIPA (RIPA:PMSF:cocktail = 100:1:1). A BCA protein kit (Thermofisher, Waltham, MA, USA) was employed to determine protein concentrations. Equal amounts of denatured proteins were then separated by 10% SDS-polyacrylamide gels and electrophoretically transferred to polyvinylidene difluoride (PVDF) membranes (Millipore, Billerica, MA, USA). They were incubated with 0.1% Tween 20 (TBS-T) and 5% skimmed milk of Tris-buffered saline at room temperature for 2 h. After blocking of non-specific binding to the membranes, the PVDF membrane was incubated with specific primary antibodies overnight at 4 °C. It was then washed with TBST and incubated with secondary antibody (Anti-rabbit IgG-HRP, 1:5000) for 2 h at room temperature. The membrane signals were analyzed by ChemiDoc™Touch Imaging System (Bio-Rad Laboratories, Hercules, CA, USA). GAPDH was used as a housekeeping protein.

#### 3.2.8. Animal Experiments

SPF BALB/c mice (male, weight 18–22 g) were purchased from Beijing Weitong Lihua Experimental Animal Technology Co., LTD., with the production license number of experimental animal SCXK (Beijing) 2021-0006. All the animals were fed adaptively for 7 days under laboratory conditions, and they could drink and eat freely. The animals were kept in a 12 h light-dark cycle (Humidity: 40–60%; Temperature: 18–24 °C). All laboratory operations and procedures were in accordance with animal testing regulations.

Sixty BALB/c mice were randomly divided into six groups (10 mice/group): control group, model group, dexamethasone group (5 mg·kg^−1^), low (**5d**, 5 mg·kg^−1^), medium (**5d**, 10 mg·kg^−1^) and high (**5d**, 20 mg·kg^−1^) dose groups. The control group was not injected with LPS, and the remaining **5d** groups were intraperitoneally injected with LPS (4 mg·kg^−1^) to make endotoxemia. The control group and model group were intraperitoneally injected with only soybean oil, and the other groups were injected with **5d** at 0, 5 and 10 h, respectively. Blood was sampled from rat eyes and routine blood examinations were detected after 2 h administration. The blood was soaked at 37 °C for 2 h and centrifuged at 3500 r·min^−1^ for 15 min. The serum was extracted and stored at −80 °C. Lung and kidney tissues of the mice were collected and then fixed with 4% PFA.

#### 3.2.9. Data Analysis

All experiments were repeated at least three times. The analysis of statistical significance was performed utilizing the GraphPad Prism 7.0 Software (GraphPad Software, San Diego, CA, USA). A one-way ANOVA with Dunnett’s multiple comparison test was used to perform group comparisons. A *p*-value lower than 0.05 (*p* < 0.05) was used for significant differences.

## 4. Conclusions 

In summary, a series of novel benzofuran-tetrazole hybrids have been designed, synthesized and characterized as novel NSAIDs anti-inflammatory candidate compounds. Among these compounds, compound **5d** had low cytotoxicity (IC_50_ > 80 μM) and the strongest inhibitory effect on NO (EC_50_ = 52.23 ± 0.97 μM). In in vitro cell experiments, compound **5d** could significantly inhibit the phosphorylation levels of IKKα/IKKβ, IKβα, P65, ERK, JNK and P38 in the MAPK/NF-κB signaling pathway and down-regulate the secretion of pro-inflammatory factors such as NO, COX-2, TNF-α and IL-6. In in vivo mice experiments, compound **5d** could regulate the involvement of neutrophils, leukocytes and lymphocytes in anti-inflammatory processes in endotoxemic mice, and significantly reduce the expression of IL-1β, TNF-α, IL-6 and other pro-inflammatory factors in serum and some tissues. The above in vitro and in vivo results indicated that compound **5d** could inhibit the entry of nuclear factor NF-κB/p65 into the nucleus, thereby blocking the activation of the MAPK/NF-κB signaling pathway, and thus demonstrating good potential as a new non-steroidal anti-inflammatory lead compound.

## Data Availability

We have presented all our main datas in the form of tables and figures. CCDC 2184860 contain supplementary crystallographic datas for compound **4d**. These datas can be obtained free of charge via http://www.ccdc.cam.ac.uk/conts/retrieving.html or the Cambridge Crystallographic Data Centre, 12 Union Road, Cambridge CB2 1EZ, UK; fax: (+44) 1223-336-033; or e-mail: deposit@ccdc.cam.ac.uk.
